# Midlife Metabolic Syndrome as Mediator Between Early-Life Insulin Resistance and Later-Life Cardiovascular Diseases

**DOI:** 10.1016/j.jacadv.2026.103419

**Published:** 2026-11-04

**Authors:** Chuyue Wu, Nathan D. Wong, Liwei Chen, Wenjun Fan, Marissa J. Seamans, Hua Zhou, Roch A. Nianogo

**Affiliations:** a Department of Epidemiology, Fielding School of Public Health, University of California, Los Angeles (UCLA), Los Angeles, California, USA; b Mary and Steve Wen Cardiovascular Division, Department of Medicine, University of California, Irvine, California, USA; c Department of Epidemiology and Biostatistics, Joe C. Wen School of Population & Public Health, University of California, Irvine, California, USA; d Department of Epidemiology, UCLA Fielding School of Public Health, Los Angeles, California, USA; e California Center for Population Research, UCLA, Los Angeles, California, USA

**Keywords:** atherosclerotic cardiovascular disease, insulin resistance, mediation analysis, metabolic syndrome

## Abstract

**Background:**

The cardiovascular-kidney-metabolic syndrome framework describes progression from early metabolic dysfunction to clinical cardiovascular disease. Insulin resistance (IR) in young adulthood may contribute to later atherosclerotic cardiovascular disease (ASCVD), but the extent to which this association is mediated through midlife metabolic syndrome (MetS) remains unclear.

**Objectives:**

We aimed to evaluate the association between young-adulthood IR and later-life ASCVD and quantify mediation through midlife MetS.

**Methods:**

We analyzed 3,622 Black and White participants in the Coronary Artery Risk Development in Young Adults study who were aged 18 to 30 years at baseline and free of ASCVD through year 20. IR was defined as a Homeostatic Model Assessment of Insulin Resistance value at or above the 75th percentile (≥2.67). MetS was assessed at year 20, and ASCVD events were ascertained through year 35. Parametric g-computation with pooled logistic regression estimated total and mediated effects on cumulative ASCVD risk at the end of follow-up, accounting for midlife lifestyle factors as exposure-induced mediator-outcome confounders.

**Results:**

At baseline, 907 of 3,622 participants (25%) had IR. During a median follow-up of 14 years (Q1-Q3: 14-14 years) after year 20, 143 of 3,622 (3.9%) participants experienced an ASCVD event. Young-adulthood IR was associated with a 1.50-fold higher ASCVD risk at the end of follow-up (95% CI: 1.13-2.01), with 63.4% of the association mediated through midlife MetS (indirect risk ratio: 1.29; 95% CI: 1.13-1.48).

**Conclusions:**

Young-adulthood IR was associated with higher later-life ASCVD risk, with a substantial proportion mediated through midlife MetS. These findings suggest early identification of IR and prevention of progression to MetS may help reduce long-term cardiovascular risk.

Atherosclerotic cardiovascular disease (ASCVD), including coronary heart disease, stroke, and peripheral artery disease, remains the leading cause of morbidity and mortality in the United States.^1^ Atherosclerosis develops progressively over decades, beginning with metabolic and vascular abnormalities long before clinical cardiovascular events occur.^2^ This life-course process is consistent with the cardiovascular-kidney-metabolic (CKM) syndrome framework, which describes progression from early metabolic dysfunction to clustered cardiometabolic risk factors, subclinical cardiovascular disease (CVD), and ultimately a clinically manifested CVD.^3^ Identifying modifiable metabolic abnormalities early in life may therefore provide opportunities to recognize and interrupt adverse CKM trajectories before ASCVD develops.

Insulin resistance (IR), characterized by impaired cellular responsiveness to insulin, commonly emerges in young adulthood and is closely related to excess adiposity and other metabolic disorders.^4^, ^5^ IR also contributes to endothelial dysfunction, oxidative stress, and systemic inflammation, mechanisms that may initiate vascular injury before conventional cardiovascular risk factors become apparent.^6^ Thus, IR in young adulthood may represent an early metabolic abnormality along the CKM continuum and may identify individuals at increased risk of long-term CVDs.

As individuals transition from young adulthood into midlife, persistent IR may contribute to the development of metabolic syndrome (MetS), a clustering of central obesity, elevated blood pressure, hyperglycemia, and dyslipidemia.^7^, ^8^, ^9^ Within the CKM framework, this transition may represent progression from an early metabolic abnormality to a more advanced state characterized by multiple established cardiometabolic risk factors. Midlife MetS may subsequently accelerate atherosclerosis and increase the risk of clinical ASCVD later in life. IR may also influence ASCVD through pathways not captured by MetS, including chronic inflammation, oxidative stress, endothelial dysfunction, and the direct vascular effects of hyperinsulinemia. Midlife lifestyle factors, including diet and physical activity, may further shape these pathways because they may be influenced by earlier IR while also affecting the development of MetS and subsequent ASCVD risk.^10^

Most prior studies have examined IR and MetS as separate cardiovascular risk factors rather than evaluating their sequential roles within a life-course CKM pathway.^11^ Young adulthood represents an important period for identifying IR before cardiometabolic risk factors become firmly established. Midlife is another pivotal period during which early IR may progress to MetS and substantially increase long-term cardiovascular risk.^12^ Although the CKM framework emphasizes this progressive course, the extent to which midlife MetS mediates the association between young-adulthood IR and later-life ASCVD remains unclear.

To address these gaps, we conducted a longitudinal mediation analysis in the CARDIA (Coronary Artery Risk Development in Young Adults) study, with IR assessed in young adulthood at ages 18 to 30 years, MetS assessed in midlife at ages 38 to 50 years, and incident ASCVD ascertained in later life. Our objective was to evaluate the association between young-adulthood IR and cumulative ASCVD risk by the end of follow-up and to quantify the extent to which this association was mediated through midlife MetS. We accounted for midlife diet and physical activity as exposure-induced confounders of the MetS-ASCVD relationship. By evaluating this life-course sequence, our study examines how young-adulthood IR may contribute to progression along the CKM continuum and identifies potential opportunities for earlier ASCVD prevention. The hypothesized causal pathways are illustrated in the directed acyclic graph in Supplemental Figure 1.

## Methods

### Study design, setting, and participants

We used data from the CARDIA study, a multicenter, population-based, prospective cohort study designed to examine cardiovascular risk development from young adulthood. At baseline (year 0; 1985-1986), 5,113 Black and White adults aged 18 to 30 years were recruited from 4 U.S. field centers and followed through periodic in-person examinations and ongoing surveillance for clinical events. Institutional Review Boards at all participating centers approved the study, and participants provided written informed consent.

The analytical sample included participants with baseline insulin and glucose measurements. A total of 1,359 participants without these measurements were excluded. An additional 132 participants with ≤20 years of follow-up were excluded to preserve the temporal ordering of the exposure, mediator, and outcome. After exclusions, 3,622 participants were included in the analysis (Figure 1). Baseline characteristics of the included and excluded CARDIA participants are presented in Supplemental Table 1.

**Figure 1:**
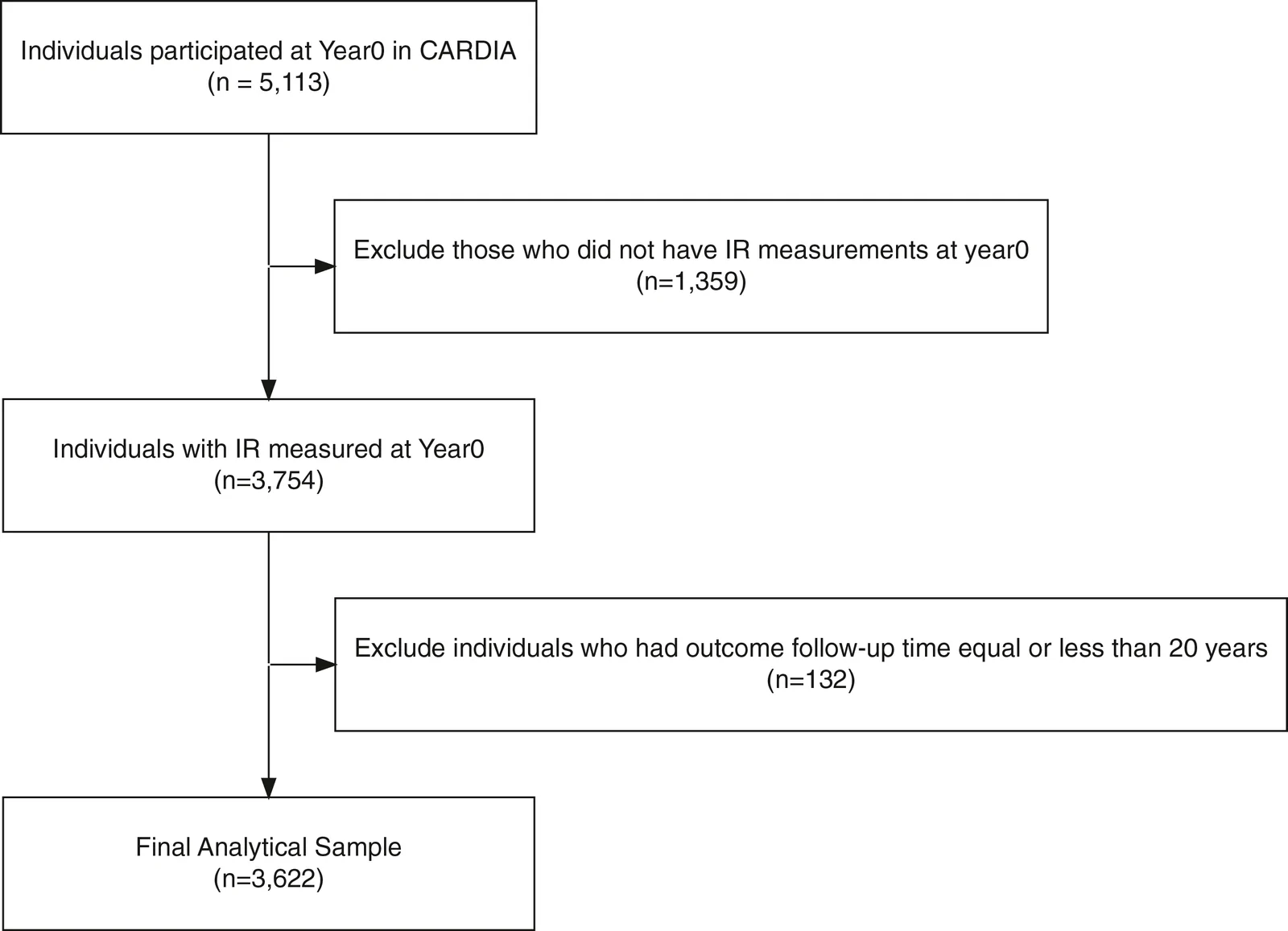
**Study Sample Flow Diagram** CARDIA = Coronary Artery Risk Development in Young Adults; IR = insulin resistance.

### Variables

#### Exposure (X): IR at year 0

IR was assessed at baseline, when participants were aged 18 to 30 years. After a 12-hour fast, plasma glucose was measured using the hexokinase ultraviolet method, and insulin was measured by radioimmunoassay. The Homeostatic Model Assessment of Insulin Resistance (HOMA-IR) was calculated as fasting insulin in μU/mL multiplied by fasting glucose in mg/dL and divided by 405.^13^ IR was defined as a HOMA-IR value at or above the 75th percentile of the analytical population, corresponding to a cutoff of 2.67.^14^

#### Mediator (M): MetS at year 20

MetS was assessed at year 20, when participants were aged 38 to 50 years, using harmonized criteria.^15^ MetS was defined as the presence of at least 3 of the following criteria: 1) waist circumference ≥102 cm in men or ≥88 cm in women; 2) triglycerides ≥150 mg/dL; 3) high-density lipoprotein cholesterol <40 mg/dL in men or <50 mg/dL in women; 4) systolic blood pressure ≥130 mm Hg, diastolic blood pressure ≥85 mm Hg, or the use of antihypertensive medication; and 5) fasting glucose ≥100 mg/dL or the use of glucose-lowering medication.

#### Outcome (Y): Fatal and nonfatal incident ASCVD after year 20

Participants were followed from year 20 through year 35 for the first fatal or nonfatal ASCVD event. ASCVD included coronary heart disease, ischemic stroke, transient ischemic attack, peripheral artery disease, and ASCVD-related death. ASCVD-related death was defined as death in which the first-listed underlying cause of death was ischemic heart disease (International Classification of Diseases-9th Revision [ICD-9] codes 410-414), cerebrovascular disease (ICD-9 codes 430-438), or generalized atherosclerosis (ICD-9 code 440). Events were identified by the CARDIA Endpoints Surveillance and Adjudication Subcommittee using standardized procedures involving study examinations, annual follow-up contacts, medical record review, and linkage to the National Death Index.^16^ Follow-up began at year 20, when MetS was assessed, and continued until the first ASCVD event, loss to follow-up, or the end of year 35.

#### Baseline confounders (C) at year 0

Information on age, sex, race, education, marital status, cigarette smoking, diet quality, physical activity, family history of ASCVD, and prevalence of MetS and diabetes was collected at baseline through structured interviews, self-administered questionnaires, and clinical examinations. Education was recorded as years completed and categorized as “high school or less” for ≤12 years. Cigarette smoking was classified as current vs noncurrent, and lifetime pack-years were calculated by multiplying the total number of years smoked by the average number of cigarettes smoked per day and dividing the product by 20 cigarettes per pack. Physical activity was measured using the validated CARDIA Physical Activity History questionnaire, which assesses 13 activity types during the previous year.^17^, ^18^ Physical activity scores, expressed in exercise units, reflect activity frequency, intensity, and duration. A score of at least 300 exercise units corresponds approximately to meeting U.S. physical activity guidelines of at least 150 minutes per week of moderate-intensity activity.^19^, ^20^, ^21^ Diet quality was assessed using an interviewer-administered, validated diet history capturing dietary intake during the preceding month. Diet quality was quantified using the A Priori Diet Quality Score, a quintile-based index incorporating 20 beneficial and 13 adverse food groups. The theoretical maximum score was 132, with higher scores indicating healthier dietary patterns.^22^, ^23^ Family history of ASCVD was defined as a self-reported parental history of heart attack or stroke. Baseline MetS was assessed using the harmonized definition,^15^ and baseline diabetes status was self-reported.

#### Exposure-induced confounders (L) at year 20

Diet quality and physical activity were reassessed at year 20. Physical inactivity was defined as fewer than 300 exercise units, and adverse diet quality was defined as an A Priori Diet Quality Score below 66, corresponding to 50% of the maximum possible score. A midlife lifestyle-risk indicator was defined as the presence of both physical inactivity and adverse diet quality.

### Handling of missing data

Baseline missingness was <1%, whereas approximately 20% of year 20 MetS and lifestyle variables were missing. Missingness patterns for variables measured at baseline and year 20 are presented in Supplemental Figure 2. We used MICE (multiple imputation by chained equations) under a missing-at-random assumption.^24^, ^25^ To improve prediction of missing values and make use of the longitudinal structure of CARDIA, the imputation models incorporated all available observed values of relevant variables across study examinations at year 0, 2, 5, 7, 10, 15, 20, 25, and 30, including IR, MetS, ASCVD, and measured covariates. Twenty imputed data sets were generated, and estimates were pooled using Rubin’s rules.^25^

### Statistical analysis

We used the parametric g-computation^26^, ^27^ adapted for survival outcomes to estimate the association between young-adulthood IR and later-life ASCVD and the extent to which this association was mediated through midlife MetS. Pooled logistic regression was used as a discrete-time survival model, with participants contributing person-time from year 20 until ASCVD, censoring, or the end of follow-up.^28^ The discrete-time data structure used 1-year intervals over 15 years of follow-up after year 20. Follow-up time was indexed from 1 to 15, permitting estimation of year-specific cumulative risks and corresponding effect measures at each follow-up year. The time-averaged effect was calculated as the average of the 15 year-specific effects, and the end-of-follow-up effect was defined as the effect at follow-up year 15.^29^

Because midlife lifestyle factors may be influenced by young-adulthood IR and may confound the association between MetS and ASCVD, we used a path-specific mediation approach that allows for exposure-induced mediator-outcome confounding.^30^, ^31^, ^32^ The total effect (TE) of IR on ASCVD was decomposed into: 1) a direct effect not operating through either midlife MetS (M) or lifestyle (L), that is, $E_{X\to Y}$; 2) a path-specific indirect effect operating through midlife MetS only, that is, $E_{X\to M\to Y}$; and 3) an indirect effect operating through midlife lifestyle, with or without MetS, that is, $E_{X\to LY}$.^33^, ^34^, ^35^ Time-averaged effects over 15-year follow-up and the end-of-follow-up effects were estimated on the risk-difference and risk-ratio scales using the parametric g-computation with pooled logistic regression. TE was calculated as the sum of the direct and path-specific indirect effects. The proportion mediated through MetS was calculated as the MetS-specific indirect effect divided by the TE.^28^, ^34^, ^35^, ^36^, ^37^ To account for uncertainty from both sampling variability and multiple imputation, 1,000 bootstrap samples were generated within each imputed data set, and estimates were pooled across imputations using Rubin’s rules to obtain 95% CI.^25^, ^38^ Detailed model specifications and instructions for step-by-step implementation of the parametric g-computation are provided in Supplemental Section 1 and Supplemental Section 2, respectively. The decomposition of the TE into the direct and path-specific indirect effects, along with the calculation of the proportion mediated, is presented in Supplemental Table 2. All analyses were performed using R, version 4.5.0 (R Foundation for Statistical Computing).

### Sensitivity analyses

As sensitivity analyses, we repeated the primary mediation analysis using 2 broader CARDIA adjudicated clinical outcomes. Fatal or nonfatal cardiovascular-renal disease (CVRD) included coronary heart disease (myocardial infarction, acute coronary syndrome, or coronary heart disease death), stroke, transient ischemic attack, hospitalization for heart failure, peripheral arterial disease requiring intervention, end-stage renal disease, and cardiovascular- or renal-related death.^39^ Fatal or nonfatal CVD included adjudicated coronary heart disease, stroke or transient ischemic attack, heart failure, peripheral arterial disease, and cardiovascular death according to CARDIA endpoint definitions.^16^, ^40^ For each outcome, follow-up began at year 20 and continued until the first corresponding event, loss to follow-up, or the end of year 35. The same parametric g-computation and path-specific mediation framework used in the primary analysis was applied.

In addition, we conducted a complete-case analysis restricted to participants with complete data on all variables included in the mediation analysis to assess the robustness of the findings from the multiple-imputation analysis.

## Results

### Baseline (year 0) participant characteristics by IR status

Compared with excluded participants, included participants were slightly older, less likely to be non-Hispanic Black or current smokers, had higher educational attainment, and reported healthier diet quality (Supplemental Table 1).

Among 3,622 CARDIA participants included in the analytical sample, the mean age at baseline was 25.0 years (SD: 3.6), 1,620 of 3,622 (45%) were men, and 1,705 of 3,622 (47%) identified as non-Hispanic Black. Overall, 907 of 3,622 participants (25%) had IR, defined as HOMA-IR ≥2.67. Compared with participants without IR, those with IR were more likely to be non-Hispanic Black (555 of 907 [61%] vs 1,150 of 2,715 [42%]), to have a parental history of heart attack or stroke (213 of 907 [23%] vs 487 of 2,715 [18%]), and to have lower physical activity (median: 311 [Q1-Q3: 163-517] vs 391 [Q1-Q3: 217-606]) and diet quality scores (mean: 59 [SD: 11] vs 62 [SD: 12]). Baseline MetS (70 of 907 [7.7%] vs 12 of 2,715 [0.4%]) and diabetes (25 of 907 [2.8%] vs 8 of 2,715 [0.3%]) were also more prevalent among participants with IR. During a median follow-up of 14 years (Q1-Q3: 14-14 years) after year 20, 143 of 3,622 participants (3.9%) experienced an ASCVD event, including 53 of 907 (5.8%) among participants with IR and 90 of 2,715 (3.3%) among those without IR (Table 1).

**Table 1:** Baseline (Year 0) Characteristics of Study Population in CARDIA Study (N = 3,622)

	Young Adulthood (Year 0) (18-30 Years Old)		
	Total (N = 3,622)	Without IR^a^ (n = 2,715)	With IR^a^ (n = 907)
Age, y, mean (SD)	25.0 (3.6)	25.1 (3.5)	24.6 (3.8)
Male, n (%)	1,620 (45)	1,211 (45)	409 (45)
Non-Hispanic Black, n (%)	1,705 (47)	1,150 (42)	555 (61)
Family history of ASCVD, n (%)	700 (19)	487 (18)	213 (23)
High school or less education, n (%)	1,305 (36)	951 (35)	354 (39)
Unmarried, n (%)	2,775 (77)	2,110 (78)	665 (73)
Current smokers, n (%)	996 (27)	792 (29)	204 (22)
Life time package years among smokers, median (Q1-Q3)	3.5 (1.1-7.5)	3.5 (1.2-7.5)	3.1 (0.8-7.8)
Physical activity score, median (Q1-Q3)	370 (204-587)	391 (217-606)	311 (163-517)
Dietary score, mean (SD)	61 (12)	62 (12)	59 (11)
Fasting insulin level, μU/mL, mean (SD)	11.5 (5.9)	9.2 (2.2)	18.2 (7.9)
Fasting glucose level, mg/dL, mean (SD)	82 (11)	80 (8)	88 (16)
HOMA-IR, median (Q1-Q3)	2.0 (1.6-2.7)	1.8 (1.5-2.2)	3.3 (2.9-4.2)
MetS^b^, n (%)	82 (2.3)	12 (0.4)	70 (7.7)
Diabetes, n (%)	33 (0.9)	8 (0.3)	25 (2.8)
Follow-up years, median (Q1-Q3)	14 (14-14)	14 (14-14)	14 (14-14)
Incident ASCVD followed up to 15 y, n (%)	143 (3.9)	90 (3.3)	53 (5.8)

Values are mean (SD) for normally distributed variables, median (Q1-Q3) for skewed variables, and n (%) for categorical variables. Missingness was <1% for all variables.

ASCVD = atherosclerotic cardiovascular disease; CARDIA = Coronary Artery Risk Development in Young Adults; HOMA-IR = Homeostatic Model Assessment of Insulin Resistance; IR = insulin resistance; MetS = metabolic syndrome.

a IR was defined as a HOMA-IR value at or above the 75th percentile of the analytical population, corresponding to a cutoff of 2.67.

b MetS was defined by the harmonized criteria^15^ as the presence of 3 or more of the 5 components.

### Total and path-specific effects of young-adulthood IR on ASCVD

Young-adulthood IR was associated with higher later-life ASCVD risk (Table 2). Averaged across follow-up years, the TE corresponded to 106 additional ASCVD events per 10,000 participants (95% CI: 22-190) and a 1.53-fold higher risk of ASCVD (risk ratio [RR]: 1.53; 95% CI: 1.06-2.20). At the end of follow-up, the TE was 191 additional events per 10,000 participants (95% CI: 47-335) and a 1.50-fold higher risk of ASCVD (RR: 1.50; 95% CI: 1.13-2.01).

**Table 2:** Decomposition of the Total Effect of Young-Adulthood IR on Later-Life ASCVD Risk, Averaged Across Follow-Up Years and at the End of Follow-Up, in the CARDIA Study (N = 3,622)

	Effect Decomposition	RD, per 10,000 (95% CI)	RR (95% CI)
Time-averaged effects	Total effect	106 (22-190)	1.53 (1.06-2.20)
	IR → ASCVD^a^	32 (−44 to 109)	1.17 (0.78-1.74)
	IR → MetS → ASCVD^b^	72 (35-108)	1.30 (1.13-1.49)
	IR → Lifestyle → ASCVD & IR → Lifestyle → MetS → ASCVD^c^	2 (−7 to 12)	1.01 (0.98-1.04)
	% Mediated^d^	67.9%	61.9%
End of follow-up effects	Total effect	191 (47-335)	1.50 (1.13-2.01)
	IR → ASCVD^a^	61 (−70 to 191)	1.15 (0.84-1.59)
	IR → MetS→ ASCVD^b^	127 (62-191)	1.29 (1.13-1.48)
	IR → Lifestyle → ASCVD & IR → Lifestyle → MetS → ASCVD^c^	4 (−12 to 20)	1.01 (0.98-1.04)
	% Mediated^d^	66.5%	63.4%

Denotations: IR: insulin resistance at year 0; Lifestyle: adverse diet quality and low physical activity at year 20; MetS: metabolic syndrome at year 20; ASCVD: incident ASCVD followed up to 15 years after year 20 (median: 14 years; Q1-Q3: 14-14 years).

RD = risk difference; RR = risk ratio; other abbreviations as in Table 1.

a IR → ASCVD: direct effect of IR on ASCVD through pathways involving neither Lifestyle nor MetS.

b IR → MetS → ASCVD: effect mediated through MetS only, not involving Lifestyle.

c IR → Lifestyle → ASCVD & IR → Lifestyle → MetS → ASCVD: effect through Lifestyle, with or without MetS.

d % Mediated: Proportion mediated through MetS is calculated as $\left({\text{RD}}_{\text{IR}\;\to \;\text{MetS}\to \;\text{ASCVD}\;}/{\text{RD}}_{Total\;Effect}\right)\times 100\%$ in RD scale and as $\log\left({\text{RR}}_{\text{IR}\;\to \;\text{MetS}\to \;\text{ASCVD}\;}\right)/\log\left({\text{RR}}_{\text{Total}\;\text{Effect}}\right)\times 100\%$ in RR scale.

The path-specific indirect effect through midlife MetS accounted for a substantial proportion of the total association between young-adulthood IR and later ASCVD. Averaged across follow-up, the MetS-mediated pathway was associated with 72 additional ASCVD events per 10,000 participants (95% CI: 35-108) and an RR of 1.30 (95% CI: 1.13-1.49), corresponding to 67.9% of the TE on the risk-difference scale and 61.9% on the risk-ratio scale. At the end of follow-up, the MetS-mediated effect was 127 additional events per 10,000 participants (95% CI: 62-191) with an RR of 1.29 (95% CI: 1.13-1.48), accounting for 66.5% and 63.4% of the TE on the risk-difference and risk-ratio scales, respectively.

By contrast, the direct pathway not operating through midlife MetS or lifestyle was smaller and imprecisely estimated. At the end of follow-up, the direct effect was 61 additional events per 10,000 participants (95% CI: −70 to 191) with an RR of 1.15 (95% CI: 0.84-1.59). The pathway through midlife adverse diet quality and low physical activity, with or without subsequent MetS, contributed minimally to the overall association (risk difference: 4 per 10,000; 95% CI: −12 to 20; RR: 1.01; 95% CI: 0.98-1.04). The overall life-course pathway and main findings are summarized in the Central Illustration.

**Central Illustration:**
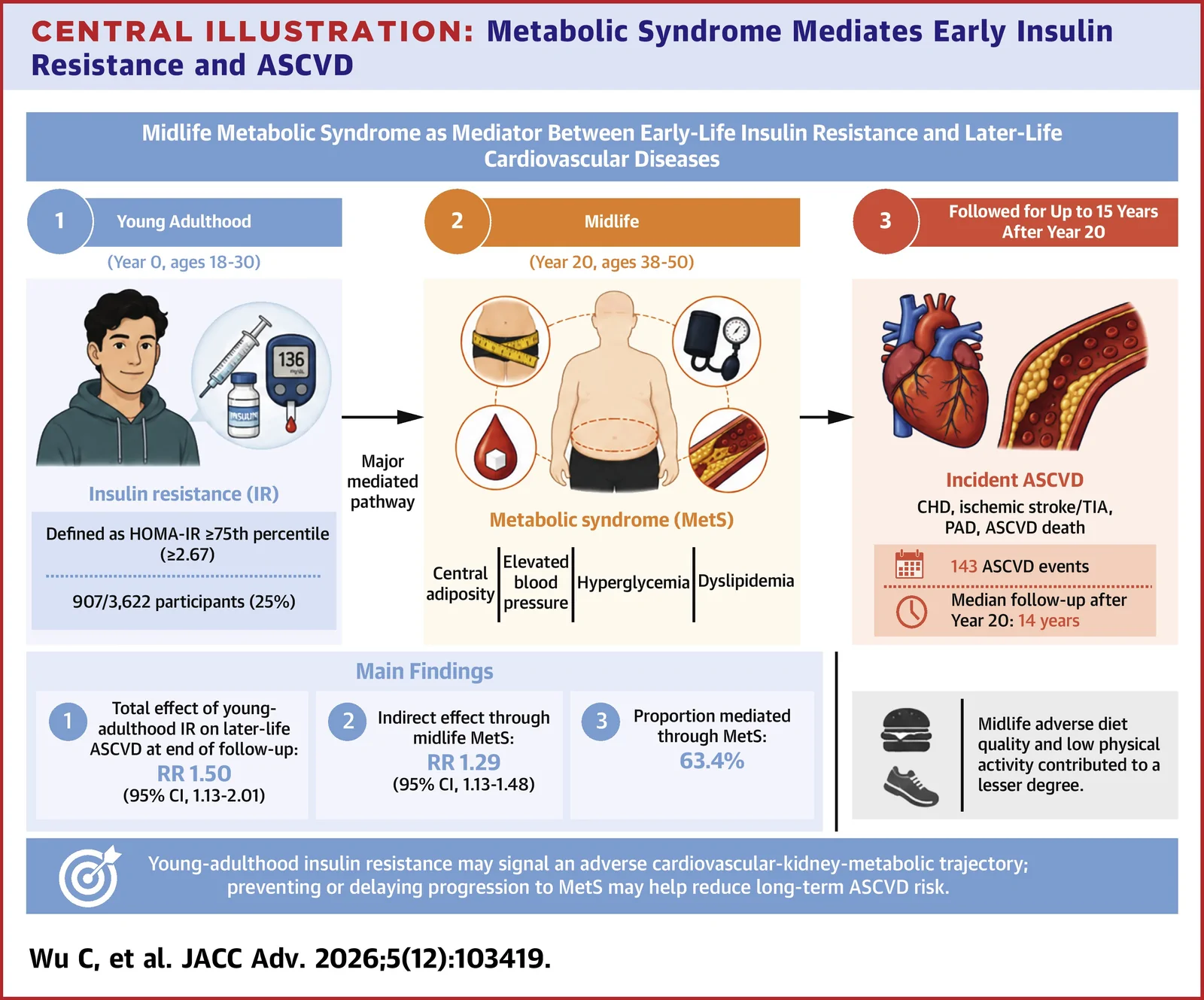
**Metabolic Syndrome Mediates Early Insulin Resistance and ASCVD** Young-adulthood IR, defined as HOMA-IR at or above the 75th percentile (≥2.67), was associated with higher risk of later-life atherosclerotic cardiovascular disease (ASCVD). MetS, characterized by central adiposity, elevated blood pressure, hyperglycemia, and dyslipidemia, represented the major mediated pathway linking early IR to subsequent ASCVD. By the end of follow-up, the total effect of young-adulthood IR on later-life ASCVD was RR = 1.50 (95% CI: 1.13-2.01), while the indirect effect through midlife MetS was RR = 1.29 (95% CI: 1.13-1.48), corresponding to 63.4% mediation on the RR scale. The pathway through adverse diet quality and low physical activity contributed to a lesser degree. These findings suggest that young-adulthood IR may signal an adverse cardiovascular-kidney-metabolic trajectory and that preventing or delaying progression to MetS may help reduce long-term ASCVD risk. ASCVD = atherosclerotic cardiovascular disease; CHD = coronary heart disease; HOMA-IR = Homeostatic Model Assessment of Insulin Resistance; IR = insulin resistance; MetS = metabolic syndrome; PAD = peripheral artery disease; RR = risk ratio; TIA = transient ischemic attack.

### Sensitivity analyses

Sensitivity analyses using the broader CVRD and CVD outcomes yielded findings consistent with those of the primary ASCVD analysis. At the end of follow-up, young-adulthood IR was associated with higher risks of CVRD (RR: 1.60; 95% CI: 1.25-2.05) and CVD (RR: 1.73; 95% CI: 1.34-2.23). The MetS-specific indirect effects were RR = 1.22 (95% CI: 1.10-1.36) for CVRD and RR = 1.21 (95% CI: 1.09-1.35) for CVD, corresponding to 42.6% and 34.5% of the TEs mediated through midlife MetS, respectively. The pathway through midlife adverse diet quality and low physical activity contributed minimally to both outcomes (Figures 2 and 3, Supplemental Table 3).

**Figure 2:**
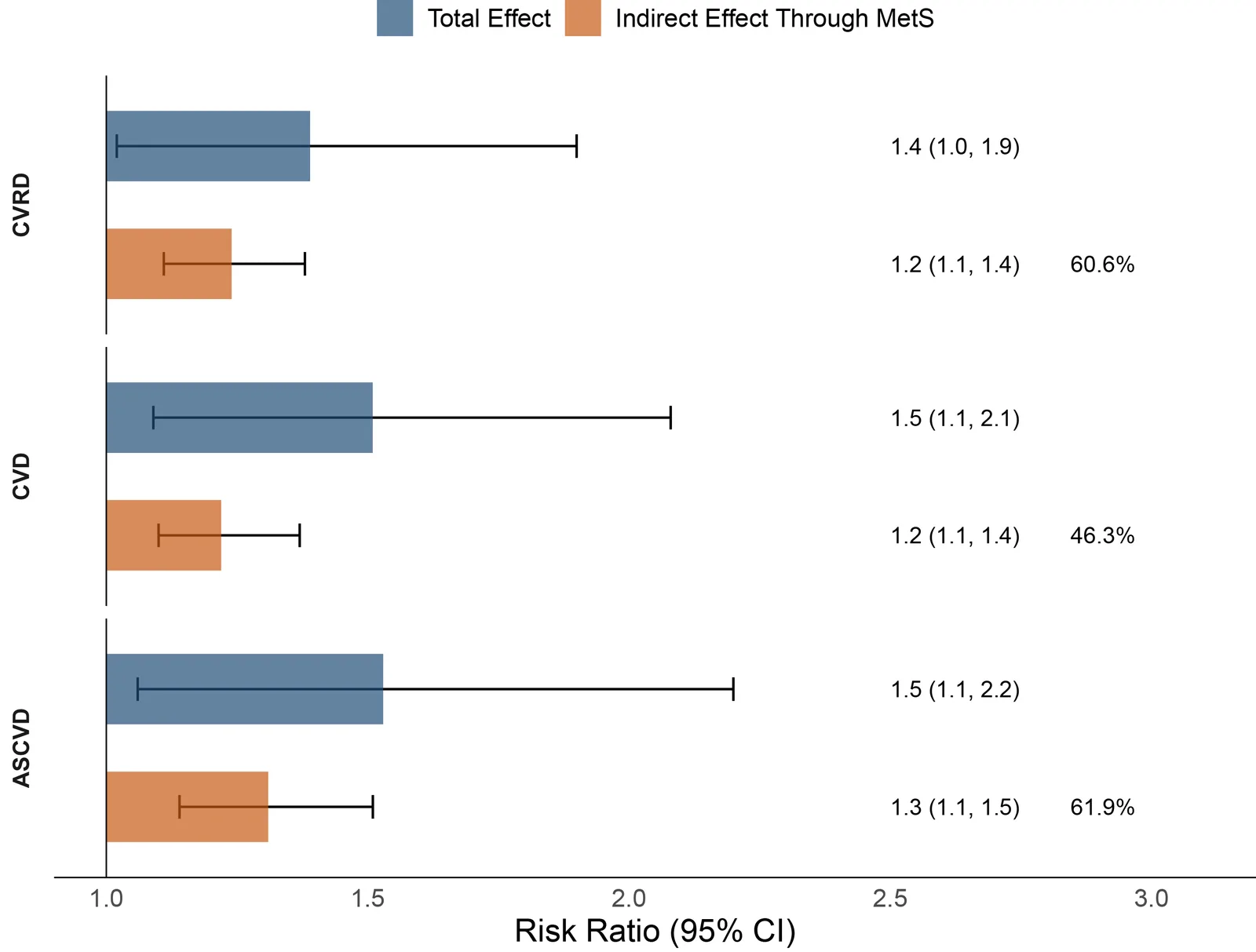
**15-Year Time-Averaged Total and MetS-Mediated Effects of Young-Adult Insulin Resistance on Cardiovascular Outcomes** Fifteen-year time-averaged total and MetS-specific indirect effects of young-adulthood insulin resistance on later-life CVRD, CVD, and ASCVD events in the CARDIA Study, presented as risk ratios with 95% CIs. Percentages indicate the proportion of the total effect mediated through the MetS-specific indirect pathway. ASCVD = atherosclerotic cardiovascular disease; CARDIA = Coronary Artery Risk Development in Young Adults; CVD = cardiovascular disease; CVRD = cardiovascular-renal disease; MetS = metabolic syndrome.

**Figure 3:**
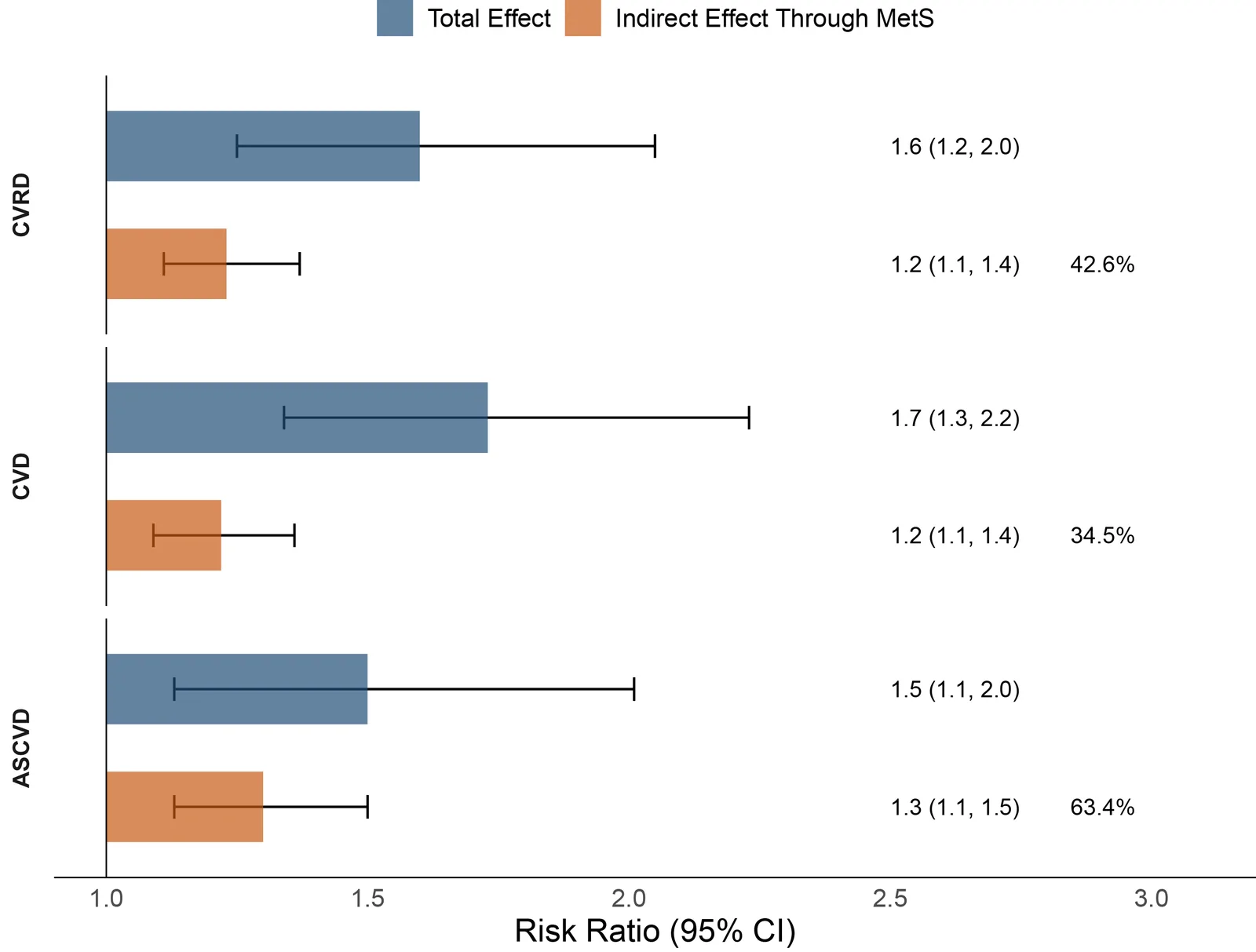
**End of Follow-Up Total and MetS-Mediated Effects of Young-Adult Insulin Resistance on Cardiovascular Outcomes** At the end of follow-up total and MetS-specific indirect effects of young-adulthood insulin resistance on later-life CVRD, CVD, and ASCVD events in the CARDIA Study, presented as risk ratios with 95% CIs. Percentages indicate the proportion of the total effect mediated through the MetS-specific indirect pathway. Abbreviations as in Figure 2.

In the complete-case analysis (n = 2,344), the MetS-specific indirect effect on ASCVD was similar in magnitude to that observed in the primary multiple-imputation analysis. At the end of follow-up, the MetS-specific indirect effect was RR = 1.34 (95% CI: 1.06-1.73), whereas the TE was RR = 1.36 (95% CI: 0.82-2.08). The wider CI for the TE reflected the reduced sample size and lower precision in the complete-case analysis (Supplemental Table 4).

## Discussion

In this longitudinal CARDIA cohort, IR in young adulthood was associated with higher ASCVD risk in later life, and a substantial proportion of this association was mediated through the development of MetS in midlife. These findings support a life-course CKM framework in which early IR may contribute to the subsequent clustering of established cardiometabolic risk factors and, ultimately, clinical CVD. They also suggest that early identification of IR and strategies to prevent or delay progression to MetS may represent important opportunities to reduce long-term cardiovascular risk.

The substantial path-specific mediation through MetS is biologically plausible because IR is a central process underlying the development and clustering of several MetS components.^41^, ^42^ Persistent IR and compensatory hyperinsulinemia can promote hyperglycemia, atherogenic dyslipidemia, elevated blood pressure, and accumulation of central adiposity.^43^ These abnormalities may reinforce one another over time and collectively increase ASCVD risk. Thus, MetS may capture the downstream cardiometabolic consequences of longstanding IR that are most directly related to clinical CVD.^44^ Within the CKM framework, the substantial mediated proportion is consistent with MetS serving as an intermediate stage between early metabolic dysfunction and later ASCVD.^3^ These findings highlight midlife MetS as an important marker of adverse CKM progression among individuals with IR earlier in adulthood.

Nevertheless, MetS did not explain the entire association between early-life IR and later-life ASCVD. IR may contribute to atherosclerosis through mechanisms not fully captured by conventional MetS criteria, including chronic inflammation, oxidative stress, endothelial dysfunction, elevated free fatty acids, and direct vascular effects of hyperinsulinemia.^45^, ^46^, ^47^, ^48^, ^49^, ^50^ In addition, the binary MetS definition may not fully reflect the severity, duration, or cumulative burden of its individual components.

Midlife diet and physical activity were incorporated as exposure-induced confounders of the MetS-ASCVD relationship to reduce bias in estimating the path-specific effect through MetS because these behaviors may be influenced by earlier metabolic dysfunctions while also affecting both MetS development and subsequent ASCVD risk. The pathway through lifestyle factors therefore reflects naturally occurring changes in diet and physical activity following young-adulthood IR, rather than the effects of a structured or intensive lifestyle intervention. Randomized trials have shown that improvements in diet, physical activity, and weight-related behaviors can improve insulin sensitivity and cardiometabolic risk factors.^51^ Accordingly, the absence of mediation through midlife lifestyle factors should not be interpreted as evidence that lifestyle modification is ineffective; rather, naturally evolving midlife diet and physical activity patterns did not explain a meaningful proportion of the IR-ASCVD association in this cohort.

These findings have potential implications for early cardiovascular prevention by suggesting that IR in young adulthood may signal an adverse CKM trajectory before conventional cardiometabolic risk factors become fully established. The substantial mediation through midlife MetS further identifies the clustering of abnormalities in adiposity, blood pressure, glucose, and lipids as a potentially important intermediate stage linking early IR to later ASCVD. Although the findings support closer cardiometabolic surveillance among young adults with IR, further research is needed to determine whether HOMA-IR improves risk prediction beyond routinely measured clinical factors, develop practical approaches for identifying IR when fasting insulin is unavailable, and test whether preventing or delaying progression to MetS reduces subsequent ASCVD risk.

### Study strengths

This study has several strengths. First, CARDIA provided repeated clinical assessments and long-term follow-up from young adulthood into later life, allowing clear temporal ordering of IR, midlife MetS, and subsequent ASCVD. Whereas previous studies have often evaluated IR and MetS as separate cardiovascular risk factors, this study examined MetS as an intermediate stage in a life-course CKM pathway, providing a life-course perspective on ASCVD prevention.^3^ Second, the parametric g-computation with pooled logistic regression adapted for survival outcomes enabled estimation of cumulative risks and corresponding risk differences and RRs for the total and path-specific effects.^29^ These measures are often more directly interpretable for public health and clinical decision-making than hazard ratios from Cox regression. By incorporating exposure-by-time interaction terms, the approach also allowed the effect of IR to vary flexibly over follow-up without imposing the proportional hazards assumption.^52^ Finally, we used a path-specific mediation approach that explicitly accounted for midlife lifestyle factors as exposure-induced mediator-outcome confounders. In this study, midlife lifestyle factors may be influenced by young-adulthood IR and also confounded the association between midlife MetS and later ASCVD. Conventional regression-based mediation methods cannot appropriately address this setting: Adjusting for lifestyle factors would block part of the causal pathway from IR through lifestyle and MetS to ASCVD, whereas omitting them would leave residual mediator-outcome confounding.^30^, ^31^, ^32^ By incorporating these factors within the path-specific effect framework, we were able to estimate direct and path-specific indirect effects while reducing bias from exposure-induced confounding.^33^

### Study limitations

Several limitations should be considered. First, HOMA-IR is an indirect measure of IR and may not correspond precisely to the gold-standard method for directly assessing insulin sensitivity using the hyperinsulinemic-euglycemic clamp.^53^ In addition, no universally accepted HOMA-IR cutoff exists. Although defining IR using the 75th percentile may limit generalizability to other populations, the resulting threshold (HOMA-IR ≥2.67) was similar to that observed among nationally representative U.S. adults without diabetes in whom the 66.7th percentile of HOMA-IR was approximately 2.6.^14^ Second, MetS was assessed at a single midlife examination. Changes in MetS status and its individual components before or after year 20 were not captured in the primary mediation analysis. Third, residual confounding by unmeasured baseline characteristics, as well as by time-varying factors beyond the measured midlife lifestyle variables, remains possible and may violate the conditional exchangeability assumptions required for causal interpretation.^54^, ^55^, ^56^ Fourth, censoring and competing events (eg, noncardiovascular deaths) were not explicitly modeled. However, follow-up was nearly complete, with a median of 14 years (Q1-Q3: 14-14 years), and only 167 of 3,622 participants (4.6%) had <14 years of follow-up without a fatal or nonfatal ASCVD event, suggesting that the potential influence of censoring or competing events was likely negligible. Fifth, participants excluded because of unavailability of baseline insulin or glucose measurements may have differed from those included in the analytical sample, which could limit the representativeness of the study population. Finally, the analytical sample was restricted to participants who remained under observation and free of ASCVD through midlife to preserve the temporal ordering of the mediator and outcome. Accordingly, the findings are generalizable primarily to individuals who survived and remained at risk of ASCVD through midlife and may not apply to those who experienced premature ASCVD events.

## Conclusions

Young-adulthood IR was associated with higher later-life ASCVD risk, and a substantial proportion of this association was mediated through the development of midlife MetS. These findings support a life-course CKM pathway linking early IR to the subsequent clustering of cardiometabolic risk factors and clinical ASCVD. They further suggest that early identification of IR and prevention or delay of progression to MetS may help reduce long-term cardiovascular risk.

Perspectives **COMPETENCY IN MEDICAL KNOWLEDGE:** IR in young adulthood was associated with higher ASCVD risk later in life, and a substantial proportion of this association was mediated through the development of MetS in midlife. These findings support a life-course CKM pathway in which early metabolic dysfunction progresses to clustered cardiometabolic risk factors and subsequent clinical ASCVD.

**TRANSLATIONAL OUTLOOK:** Early identification of IR and strategies to prevent or delay progression to MetS may represent opportunities to reduce long-term cardiovascular risk. Future studies should determine whether integrating HOMA-IR with routinely measured cardiometabolic factors improves early identification of adverse CKM trajectories and whether intervening before the development of midlife MetS can alter long-term ASCVD risk.

### Data Availability

The data supporting the findings of this study were obtained from the CARDIA study. Access to CARDIA data is available through the National Heart, Lung, and Blood Institute (NHLBI) BioLINCC repository, subject to reasonable request, data use agreements, and appropriate institutional approvals.

## Funding support and author disclosures

This work was supported by the National Institute on Minority Health and Health Disparities (K01MD014163), National Institutes of Health, Bethesda, MD. The content is solely the responsibility of the authors and does not necessarily represent the official views of the National Institutes of Health (NIH). The funder had no role in the study design, data collection and analysis, decision to publish, or preparation of the manuscript. The authors have reported that they have no relationships relevant to the contents of this paper to disclose.
