# Supplementary material for: Midlife Metabolic Syndrome as Mediator Between Early-Life Insulin Resistance and Later-Life Cardiovascular Diseases
Source: JACC Adv. 2026 Nov 4;5(12):103419. doi: 10.1016/j.jacadv.2026.103419 (PMC13753565; doi:10.1016/j.jacadv.2026.103419)
Supplement: Supplementary file 1 [file mmc1.docx]

**SUPPLEMENTARY MATERIALS**

Midlife Metabolic Syndrome as Mediator Between Early-Life Insulin Resistance and Later-life Cardiovascular Diseases

Chuyue Wu, Nathan D. Wong, Liwei Chen, Wenjun Fan, Marissa J. Seamans, Hua Zhou, Roch A. Nianogo

**eSection 1.** Model Specifications.

**eSection 2.** Parametric G-Computation Framework for Causal Mediation Analysis Using Pooled Logistic Regression with Sample R Code.

**eTable 1.** Distribution of Baseline Characteristics between Included and Excluded Participants in CARDIA.

**eTable 2.** Definitions of the Total, Direct, and Path-Specific Indirect Effects and Proportion Mediated.

**eTable 3.** Decomposition of the Total Effect of Young-Adult IR on Fatal or Nonfatal CVRD and CVD Risk, Averaged Across Follow-Up and at the End of Follow-Up, in the CARDIA Study.

**eTable 4.** Complete-Case Analysis of the Total Effect Decomposition of Young-Adult Insulin Resistance on Later-Life ASCVD Risk in the CARDIA Study (n=2,344).

**eFigure 1.** Directed acyclic graph (DAG) of analytic framework.

**eFigure 2. Missingness of Baseline and Year 20 Study Variables.**

**eSection 1.** Model Specifications.

Three regression models were specified for the parametric g-computation analysis: an outcome $Y$ model for incident ASCVD, a mediator $M$ model for midlife metabolic syndrome (MetS), and an exposure-induced mediator–outcome confounder $L$ model for the representing adverse midlife lifestyle.

Let $X$ denote insulin resistance (IR) at Year 0, $L$ denote adverse diet quality and physical inactivity at Year 20, $M$ denote MetS at Year 20, $Y_{t}$ denote incident ASCVD during follow-up interval $t$, and $C$denote the baseline covariates. Baseline covariates included age, sex, race, education, marital status, current smoking, lifetime pack-years, diet quality, physical activity, family history of ASCVD, MetS, and diabetes.

**Outcome** $\boldsymbol{Y}$ **Model**

The outcome model estimated the conditional probability of an incident ASCVD event during each follow-up interval among participants who remained event-free at the beginning of that interval. The model included young-adulthood IR, midlife MetS, adverse midlife lifestyle, linear and quadratic functions of follow-up time, and baseline covariates. Interactions between IR and time allowed the association between IR and ASCVD to vary over follow-up. A three-way interaction among IR, MetS, and adverse lifestyle, together with all corresponding lower-order terms, allowed the associations of MetS and lifestyle with ASCVD to vary according to IR and to each other.

$$logit\left[ P\left( Y_{t}=1|Y_{t-1}=0, X,M,L,C \right) \right]=\beta_{0}+\beta_{1}X+\beta_{2}t+\beta_{3}t^{2}+\beta_{4}(X\times t)+\beta_{5}(X\times t^{2})+\beta_{6}M+\beta_{7}L+\beta_{8}\left( X\times M \right)+\beta_{9}\left( X\times L \right)+\beta_{10}(M\times L)+\beta_{11}(X\times M\times L)+\beta_{12}C$$

**Mediator** $\boldsymbol{M}$ **Model**

The mediator model estimated the probability of MetS at Year 20 conditional on young-adulthood IR, adverse midlife lifestyle, and baseline covariates. The interaction between IR and adverse lifestyle allowed the association of lifestyle with MetS to differ according to baseline IR status.

$$logit\left[ P\left( M=1|X,L,C \right) \right]=\gamma_{0}+\gamma_{1}X+\gamma_{2}L+\gamma_{3}\left( X\times L \right)+\gamma_{4}C$$

**Exposure-Induced Confounder** $\boldsymbol{L}$ **Model**

The lifestyle model estimated the probability of having both adverse diet quality and physical inactivity at Year 20 conditional on young-adulthood IR and baseline covariates.

$$logit\left[ P\left( L=1|X,C \right) \right]=\delta_{0}+\delta_{1}X+\delta_{2}C$$

**eSection 2.** Parametric G-Computation Framework for Causal Mediation Analysis Using Pooled Logistic Regression with Sample R Code.

| **Parametric G-computation for time-to-event mediation analysis was implemented using the following sequential steps:** | **Sample R Code:** |
| --- | --- |
| Step 1. Data restructure. Time-to-event data were converted into a person-period format dataset, in which each participant contributed one record for each follow-up year. This data structure enabled the use of pooled logistic regression models to approximate discrete-time survival processes. |  |
| Step 2. Model specification. Pooled logistic regression models were fitted for the binary outcome (Y), and logistic models were fitted for the binary mediator (M) and binary exposure-induced confounder (L). These models were specified according to the causal structure described in **eSection 1**. |  |
| Step 3. Obtain counterfactual values of M and L. For each participant, counterfactual values of M and L were generated under both the exposed (X = 1) and unexposed (X = 0) conditions. |  |
| Step 4. Predict outcome probabilities. Using the estimated models, predicted outcome probabilities were obtained for each follow-up interval under all potential counterfactual combinations of X, M, and L. For example, $y_{X=1, m_{X=0}, l_{X=0}}$ represents the predicted outcome under exposed while fixing M and L values to those expected under unexposed. |  |
| Step 5. Estimation of causal effects. From the simulated outcome probabilities, we estimated total effects and path-specific effects at each follow-up time point. The proportion mediated through MetS was calculated as the MetS-specific indirect effect divided by the total effect. |  |
| Step 6. Summary measures. We summarized these estimated effects into: (1) end-of-follow-up effects, defined as the time-specific effects at Year 35 (i.e., 15 years after mediator assessment), and (2) time-averaged effects, defined as the average of the 15 time-specific effects across the follow-up period. These measures quantify both the long-term and average mediation effects. |  |
| Step 7. Statistical inference. Within each imputed dataset, 1000 bootstrap samples were generated to estimate effect measures and their standard errors. The resulting point estimates and standard errors from the 20 imputed datasets were then combined using Rubin’s rules to obtain the final pooled estimates and 95% confidence intervals. |  |

**eTable 1.** Distribution of Baseline Characteristics between Included and Excluded Participants in CARDIA.

| **Characteristics** | **Included**  **(n=3,622)** | **Excluded**  **(n=1,491)** | **Absolute SMD^†^** |
| --- | --- | --- | --- |
| **Age, years, mean (SD)** | 25.0 (3.6) | 24.5 (3.7) | 0.12 |
| **Male, n (%)** | 1,620 (45) | 707 (47) | 0.05 |
| **Non-Hispanic Black, n (%)** | 1,705 (47) | 932 (63) | 0.31 |
| **High School or Less Education, n (%)** | 1,305 (36) | 724 (49) | 0.26 |
| **Unmarried, n (%)** | 2,775 (77) | 1,196 (80) | 0.09 |
| **Current Smokers, n (%)** | 996 (28) | 548 (37) | 0.20 |
| **Life Time Package Years among Smokers, median (IQR)** | 3.5 (1.1, 7.5) | 3.0 (1.0, 7.0) | 0.04 |
| **Family history of ASCVD, n (%)** | 700 (19) | 295 (20) | 0.04 |
| **Physical activity score, median (IQR)** | 370 (204, 587) | 342 (178, 570) | 0.05 |
| **Dietary score, mean (SD)** | 61 (12) | 59 (11) | 0.20 |
| **MetS^*^, n (%)** | 82 (2.3) | 37 (2.7) | 0.03 |
| **Diabetes, n (%)** | 33 (0.9) | 25 (1.8) | 0.08 |

**Notes:** Values are presented as mean (SD) for normally distributed variables, median (IQR) for skewed variables, and n (%) for categorical variables. *MetS was defined by the harmonized criteria^3^ as the presence of 3 or more of the 5 components. ^†^SMDs, standardized mean differences were calculated as the difference in group means divided by the pooled standard deviation. An absolute SMD <0.10 was considered indicative of good comparability between groups.

**Abbreviations:** CARDIA, coronary artery risk development of young adults; SMD, standardized mean difference; SD, standard deviation; IQR, interquartile range; MetS, metabolic syndrome.

**eTable 2.** Definitions of the Total, Direct, and Path-Specific Indirect Effects and Proportion Mediated.

| **Measures** | **DAG** | **Expression and Interpretation** |
| --- | --- | --- |
| $E_{X\to Y}$: Direct effect of X on Y, not through either L or M |  | $\mathrm{RD}_{X\to Y}=E\left( y_{X=1, m_{X=0}, l_{X=0}} \right)-E\left( y_{X=0, m_{X=0},l_{X=0}} \right)$  $log(\mathrm{RR}_{X\to Y})=log[\frac{E\left( y_{X=1, m_{X=0}, l_{X=0}} \right)}{E\left( y_{X=0, m_{X=0},l_{X=0}} \right)}]$  Compares ASCVD risk if IR is present in young adulthood (X=1) versus absent (X=0), while holding both MetS and lifestyle fixed to the levels they would take under no IR.  How much ASCVD risk is attributable to IR itself, not operating through either MetS or lifestyle? |
| $E_{X\to M\to Y}$: Indirect effect via M only |  | $\mathrm{RD}_{X\to M\to Y}=E\left( y_{X=1, m_{X=1,l_{X=0}}, l_{X=0}} \right)-E\left( y_{X=1, m_{X=0},l_{X=0}} \right)$  $log(\mathrm{RR}_{X\to M\to Y})=log[\frac{E\left( y_{X=1, m_{X=1,l_{X=0}}, l_{X=0}} \right)}{E\left( y_{X=1, m_{X=0},l_{X=0}} \right)}]$  Compares ASCVD risk among individuals with IR, between those whose MetS status is set to what it would be under IR versus what it would be under no IR, while keeping lifestyle fixed at the no-IR level.  How much ASCVD risk would be prevented if young-adulthood IR did not cause midlife MetS? |
| $E_{X\to LY}$: Indirect effect via L, with or without M, including X→L→Y and X→L→M→Y |  | $\mathrm{RD}_{X\to LY}=E\left( y_{X=1, m_{X=1},l_{X=1}} \right)-E\left( y_{X=1, m_{X=1,l_{X=0}}, l_{X=0}} \right)$  $log(\mathrm{RR}_{X\to LY})=log[\frac{E\left( y_{X=1, m_{X=1},l_{X=1}} \right)}{E\left( y_{X=1, m_{X=1,l_{X=0}}, l_{X=0}} \right)}]$  Compares ASCVD risk among individuals with IR whose lifestyle is set to the level it would take under IR versus the level it would take under no IR, while allowing MetS to vary naturally under IR in both cases.  How much ASCVD risk is attributable to IR-induced midlife lifestyle changes, with or without MetS involvement? |
| TE: total effect |  | $\mathrm{RD}_{TE}=\mathrm{RD}_{X\to Y}+\mathrm{RD}_{X\to M\to Y}+\mathrm{RD}_{X\to LY}$  ${log(RR}_{TE})=\log\left( \mathrm{RR}_{X\to Y} \right)+\log\left( \mathrm{RR}_{X\to M\to Y} \right)+log(\mathrm{RR}_{X\to LY})$  The sum of the direct effect and all path-specific indirect effects, representing the overall effect of IR on ASCVD. |
| Proportion mediated through MetS |  | In Risk difference scale:  $\% Mediated={(RD}_{X\to M\to Y}/\mathrm{RD}_{TE})\times100\%$  In Risk ratio scale:  $\% Mediated=\log\left( \mathrm{RR}_{X\to M\to Y} \right)/\log\left( \mathrm{RR}_{\mathrm{TE}} \right)\times100\%$  The proportion of the total effect of IR on ASCVD that is explained by the indirect pathway through MetS. |

**Notes:** Abbreviations: RD, risk difference; RR, risk ratio; TE, Total Effect. % Mediated, proportion mediated. X: insulin resistance at Year 0; L: adverse diet and low physical activity at Year 20; M: metabolic syndrome at Year 20; Y: time to incident ASCVD followed up to 15 years (median: 14 years, IQR 14-14 years).

**eTable 3.** Decomposition of the Total Effect of Young-Adult IR on Fatal or Nonfatal CVRD and CVD Risk, Averaged Across Follow-Up and at the End of Follow-Up, in the CARDIA Study.

| **Outcome** | **Time** | **Effect Decomposition** | **RD, per 10,000 (95% CI)** | **RR (95% CI)** |
| --- | --- | --- | --- | --- |
| CVRD  (n=3,594) | Time-averaged Effects | Total Effect | 137 (43, 231) | 1.39 (1.02, 1.90) |
|  |  | IR → CVRD**^†^** | 58 (-30, 146) | 1.13 (0.81, 1.57) |
|  |  | IR → MetS → CVRD^‡^ | 75 (37, 114) | 1.23 (1.10, 1.37) |
|  |  | IR → Lifestyle → CVRD  &  IR → Lifestyle → MetS → CVRD^§^ | 4 (-6, 14) | 1.01 (0.98, 1.03) |
|  |  | % Mediated^*^ | 54.7% | 60.6% |
|  | End of Follow up Effects | Total Effect | 282 (121, 444) | 1.60 (1.25, 2.05) |
|  |  | IR → CVRD **^†^** | 143 (-7, 294) | 1.30 (1.00, 1.70) |
|  |  | IR → MetS → CVRD ^‡^ | 133 (65, 201) | 1.22 (1.10, 1.36) |
|  |  | IR → Lifestyle → CVRD  &  IR → Lifestyle → MetS → CVRD^§^ | 6 (-11, 24) | 1.01 (0.98, 1.03) |
|  |  | % Mediated^*^ | 47.2% | 42.6% |
| CVD  (n=3,595) | Time-averaged Effects | Total Effect | 151 (58, 244) | 1.51 (1.09, 2.08) |
|  |  | IR → CVD**^†^** | 78 (-9, 165) | 1.23 (0.88, 1.73) |
|  |  | IR → MetS → CVD^‡^ | 70 (32, 108) | 1.22 (1.09, 1.36) |
|  |  | IR → Lifestyle → CVD  &  IR → Lifestyle → MetS → CVD^§^ | 3 (-7, 13) | 1.01 (0.98, 1.03) |
|  |  | % Mediated^*^ | 46.4% | 46.3% |
|  | End of Follow up Effects | Total Effect | 310 (150, 469) | 1.73 (1.34, 2.23) |
|  |  | IR → CVD**^†^** | 180 (30, 330) | 1.42 (1.08, 1.87) |
|  |  | IR → MetS → CVD^‡^ | 125 (57, 193) | 1.21 (1.09, 1.35) |
|  |  | IR → Lifestyle → CVD  &  IR → Lifestyle → MetS → CVD^§^ | 5 (-13, 23) | 1.01 (0.98, 1.03) |
|  |  | % Mediated^*^ | 40.3% | 34.5% |

**Abbreviations:** CARDIA, coronary artery risk development in young adults; CI, confidence interval; CVRD, cardiovascular-renal disease; CVD, cardiovascular disease; IR, insulin resistance; RD, risk difference; RR, risk ratio.

**Denotations:** IR: insulin resistance at Year 0; Lifestyle: adverse diet quality and low physical activity at Year 20; MetS: metabolic syndrome at Year 20; CVRD/CVD: incident CVRD/CVD followed up to 15 years after Year 20 (median: 14 years, IQR 14-14 years).

^†^Direct effect of IR on CVRD/CVD through pathways involving neither Lifestyle nor MetS;

^‡^Effect of IR on CVRD/CVD mediated through MetS only, not involving Lifestyle;

^§^Effect of IR on CVRD/CVD through Lifestyle, with or without MetS.

^*^% Mediated: Proportion mediated through MetS is calculated as ${(RD}_{IR \to MetS\to CVRD/CVD}/\mathrm{RD}_{Total Effect})\times100\%$ in RD scale and as $\log\left( \mathrm{RR}_{IR \to MetS\to CVRD/CVD} \right)/\log\left( \mathrm{RR}_{Total Effect} \right)\times100\%$ in RR scale.

**eTable 4.** Complete-Case Analysis of the Total Effect Decomposition of Young-Adult Insulin Resistance on Later-Life ASCVD Risk in the CARDIA Study (n=2,344).

|  | **Effect Decomposition** | **RD, per 10,000 (95% CI)** | **RR (95% CI)** |
| --- | --- | --- | --- |
| **Time-averaged Effects** | Total Effect | 84 (-43, 231) | 1.54 (0.69, 2.97) |
|  | IR → ASCVD**^†^** | 12 (-101, 152) | 1.14 (0.47, 2.31) |
|  | IR → MetS → ASCVD^‡^ | 73 (15, 128) | 1.35 (1.06, 1.75) |
|  | IR → Lifestyle → ASCVD  &  IR → Lifestyle → MetS → ASCVD^§^ | -1 (-11, 18) | 0.99 (0.96, 1.07) |
| **End of Follow up Effects** | Total Effect | 137 (-75, 363) | 1.36 (0.82, 2.08) |
|  | IR → ASCVD**^†^** | 6 (-182, 220) | 1.02 (0.54, 1.62) |
|  | IR → MetS → ASCVD^‡^ | 130 (27, 230) | 1.34 (1.06, 1.73) |
|  | IR → Lifestyle → ASCVD  &  IR → Lifestyle → MetS → ASCVD^§^ | 0 (-18, 31) | 1.00 (0.96, 1.07) |

**Abbreviations:** ASCVD, atherosclerotic cardiovascular disease; CARDIA, coronary artery risk development in young adults; CI, confidence interval; IR, insulin resistance; RD, risk difference; RR, risk ratio.

**Denotations:** IR: insulin resistance at Year 0; Lifestyle: adverse diet quality and low physical activity at Year 20; MetS: metabolic syndrome at Year 20; ASCVD: incident ASCVD followed up to 15 years after Year 20 (median: 14 years, IQR 14-14 years).

^†^IR → ASCVD: direct effect of IR on ASCVD through pathways involving neither Lifestyle nor MetS;

^‡^IR → MetS → ASCVD: effect mediated through MetS only, not involving Lifestyle;

^§^IR → Lifestyle → ASCVD & IR → Lifestyle → MetS → ASCVD: effect through Lifestyle, with or without MetS.

**eFigure 1.** Directed acyclic graph (DAG) of analytic framework. The diagram illustrates the hypothesized pathways linking young-adulthood insulin resistance (IR) to later-life atherosclerotic cardiovascular disease (ASCVD). Midlife metabolic syndrome (MetS) is considered the primary mediator, while midlife lifestyle (adverse diet quality and physical inactivity) is modeled as an exposure-induced mediator–outcome confounder. In this framework: IR → ASCVD denotes the direct effect not operating through MetS or Lifestyle; IR → MetS → ASCVD denotes the effect through MetS only; IR → Lifestyle → ASCVD and IR → Lifestyle → MetS → ASCVD denote the effect through Lifestyle, with or without MetS; and the total effect (TE) is the combination of the direct and all indirect pathways. Baseline confounders (C) include age, sex, race, education, marital status, cigarette smoking, diet quality, physical activity, family history of heart attack or stroke, prevalence of metabolic syndrome, and prevalence of diabetes.

**eFigure 2. Missingness of Baseline and Year 20 Study Variables.** Bars represent the percentage of participants with missing values for each study variable measured at baseline (young adulthood, ages 18-30 years old) or Year 20 (ages 38-50 years old). Baseline variables had minimal missingness, generally less than 1%, with the exception of family history of ASCVD, which was missing for approximately 14% of participants. In contrast, Year 20 variables had greater missingness, including metabolic syndrome, physical activity, and diet score, with approximately 19%–28% of observations missing. Percentages are displayed at the end of each bar.
